# Structure prediction analysis of human core TIM23 complex reveals conservation of the protein translocation mechanism

**DOI:** 10.1002/2211-5463.13840

**Published:** 2024-06-04

**Authors:** Klaudia K. Maruszczak, Piotr Draczkowski, Artur Wnorowski, Agnieszka Chacinska

**Affiliations:** ^1^ IMol Polish Academy of Sciences Warsaw Poland; ^2^ National Bioinformatics Infrastructure Sweden, SciLifeLab Solna Sweden; ^3^ Department of Synthesis and Chemical Technology of Pharmaceutical Substances Medical University of Lublin Poland; ^4^ Department of Biopharmacy Medical University of Lublin Poland

**Keywords:** mitochondria, mitochondrial import, TIM23 complex, TIM23 structure prediction

## Abstract

The majority of mitochondrial proteins are encoded in the nucleus, translated on cytosolic ribosomes, and subsequently targeted to the mitochondrial surface. Their further import into the organelle is facilitated by highly specialized protein translocases. Mitochondrial precursor proteins that are destined to the mitochondrial matrix and, to some extent, the inner membrane, utilize translocase of the inner membrane (TIM23). This indispensable import machinery has been extensively studied in yeast. The translocating unit of the TIM23 complex in yeast consists of two membrane proteins, Tim17 and Tim23. In contrast to previous findings, recent reports demonstrate the primary role of Tim17, rather than Tim23, in the translocation of newly synthesized proteins. Very little is known about human TIM23 translocase. Human cells have two orthologs of yeast Tim17, TIMM17A and TIMM17B. Here, using computational tools, we present the architecture of human core TIM23 variants with either TIMM17A or TIMM17B, forming two populations of highly similar complexes. The structures reveal high conservation of the core TIM23 complex between human and yeast. Interestingly, both TIMM17A and TIMM17B variants interact with TIMM23 and reactive oxygen species modulator 1 (ROMO1); a homolog of yeast Mgr2, a protein that can create a channel‐like structure with Tim17. The high structural conservation of proteins that form the core TIM23 complex in yeast and humans raises an interesting question about mechanistic and functional differences that justify existence of the two variants of TIM23 in higher eukaryotes.

## Graphical abstract

The mitochondrial TIM23 translocase imports matrix and inner mitochondrial membrane‐targeted proteins. Here, we show—using computational tools—that the structural conformations of the core TIM23 complex of yeast and humans are highly similar. Our results indicate that the mechanism of protein import via the TIM23 complex is a conserved feature across eukaryotes.

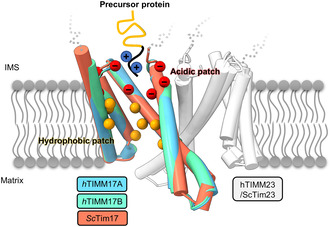

**Abbreviations**
IMS: intermembrane space
Mgr2: mitochondrial genome required
MTS: mitochondrial targeting sequence
RMSD: root mean square deviation
ROMO1: reactive oxygen species (ROS) modulator 1
TIM: translocase of the inner membrane

The vast majority of mitochondrial proteins are encoded in nuclear DNA and translated on cytosolic ribosomes. They are imported into the organelle via specialized translocation pathways [1, 2, 3]. Mitochondrial precursor proteins carry different targeting signals that guide them into one of four compartments within the organelle [4, 5]. The classical pathway involves the import of protein precursors that possess an N‐terminally localized amphipathic presequence that functions as the mitochondrial targeting sequence (MTS) [6, 7]. Such proteins are first translocated via translocase of the outer membrane (TOM) and then translocase of the inner membrane (TIM23) to the matrix or inner mitochondrial membrane.

The protein‐conducting TIM23 core consists of Tim17 and Tim23 in yeast. Both proteins contain four transmembrane domains and have similar topologies and primary sequences [8, 9]. A significant body of research demonstrated that Tim23 forms an aqueous channel either alone or by interacting with another protein to facilitate the translocation of matrix‐targeted proteins [7, 10, 11, 12, 13, 14, 15, 16]. However, the first cryogenic electron microscopy (cryo‐EM) structures of the yeast TIM23 core complex were very recently published, suggesting an architectural role of Tim23 [17, 18]. They revealed a specific back‐to‐back association of the heterodimer that is formed by Tim17 and Tim23. According to fold characteristics of the Tim17 family (Pfam PF02466), arrangement of the four transmembrane helixes induces the formation of cavities in both Tim17 and Tim23, which in their heterodimeric form are exposed toward the lipid bilayer. Recent structural data showed that the cavity that is formed by Tim17 is sufficiently large to accommodate translocating proteins [17]. The cavity that is formed by Tim23 is restricted by the inward‐tilted transmembrane helix 4 (α4). The primary role of Tim17 in protein import was also substantiated by biochemical studies [19]. Additionally, Mgr2, a small membrane‐spanning protein that is responsible for the quality control of inner membrane‐targeted proteins, was found to associate and form a channel‐like structure with Tim17 [17, 18, 20].

Biochemical characterization of the channel that is formed by Tim17 revealed specific features that are critical for its function. First, an acidic patch localized at the intermembrane space (IMS) site functions to attract positively charged presequences [19]. Second, the hydrophobic lining of the cavity that is formed by Tim17 facilitates the translocation of proteins. Third, the presence of an intramolecular disulfide bond in Tim17 was shown to be crucial for its function. Its absence significantly impaired the ability of the TIM23 complex to import its clients in yeast [21, 22].

Little is known about the structural arrangements of the TIM23 complex in human mitochondria. Human cells express two orthologs of yeast Tim17, namely TIMM17A and TIMM17B, both known to build separate pools of TIM23 complexes [23]. Here, using computational tools, we demonstrate the high similarity of human translocase to one that derives from yeast. This includes several characteristic features, such as the presence of negative charges in TIMM17 that mark the path for interactions with a precursor and disulfide bonds. Furthermore, TIMM17A and TIMM17B are alike. Our structure prediction analysis showed that the protein channel that is supported by TIMM23 and formed by transmembrane regions of TIMM17A or TIMM17B and reactive oxygen species modulator 1 (ROMO1), a human homolog of yeast Mgr2, share highly similar features. Overall, we demonstrate the conserved nature of the core TIM23 complex between yeast and humans and the large similarity of TIMM17A‐ and TIMM17B‐containing variants of human translocase.

## Materials and methods

### Gene expression data mining

Data on gene expression in cancer‐affected and non‐cancerous (normal) tissues were retrieved from the Genevestigator database (version 9.14.0) using Condition Search Tools (PMID: 19956698) [24]. For cancer cases, the following samples were excluded: metastatic samples, experimental treatment samples, primary cell cultures, pre‐cancerous lesions, and tumor microvasculature samples. Non‐cancerous (normal) samples were defined as originating from healthy controls or as histologically normal tissue adjacent to the tumor. General exclusion criteria for non‐cancerous tissues were the following: positive diseased status, experimental treatment samples, primary cell cultures, tumor bed tissue (tumor margin), and origin purely from organ muscle or mucosa. Identification numbers of datasets that were used in the analysis are provided in the Table S1.

### Multiple sequence alignments

The sequences of Tim17 from 16 different species, among which nine belonged to the Eumetazoa sub‐kingdom, were retrieved from the UniPort database (UniProt IDs of the sequences are shown in Fig. S2) [25]. The sequences were aligned using Clustal Omega with Mbed‐Like Clustering and zero combined guide‐tree/HMM iterations [26]. MView and ESprint3 were used to analyze the alignment results and for graphical representation [26, 27].

### TIM23 structure prediction

Structures of the human and yeast TIM23 core, composed of Tim23, Tim17 (TIMM17A or TIMM17B in the case of the human complex), Tim44, and Mgr2 in the yeast complex or ROMO1 in the human complex, were predicted using ColabFold (also locally as LocalColabFold) implementation of the AphaFold2.3 multimer model [28, 29, 30]. Sequences of yeast TIM23 complex components were retrieved from the UniProt database with the following IDs: yeast ScTim23 (P32897), ScTim17 (P39515), ScTim44 (Q01852), ScMgr2 (Q02889). For the prediction of human TIM23 proteins, following UniProt IDs were used: hTIMM23 (O14925), hTIMM17A (Q99595), hTIMM17B (O60830), hTim44 (O43615), hROMO1 (P60602). For each of the complexes, five models were predicted of which the top‐ranked structure (as suggested by the algorithm based on pLDDT [per residue confidence prediction], and ipTM [accuracy of the predicted interface between the subunits of the complex]) was relaxed in the AMBER force field to minimize clashes and improve local geometry. For the comparison of yeast and human Tim23‐Tim17 heterodimers and Mrg2 with ROMO1, only regions of AlphaFold structures with high prediction confidence (pLDDT ≥ 70) were used and shown in the figures. The predicted human TIM23 complex and yeast TIM23 were validated by comparing them with the published experimental cryo‐EM structure of yeast TIM23 (Protein Data Bank [PDB] accession codes 8SCX and 8E1M) [17]. For the structural analysis and comparison with the human TIM23 complex, the experimental yeast TIM23 structure was used whenever possible.

### Analysis of chemical properties of TIM23 complexes

Analyses of inter‐ and intramolecular interactions, distances, surface hydrophobicity, and electrostatic potential were performed using ucsf chimerax [31]. Contacts between yeast Tim17 and Mgr2 and human TIMM17A/B and ROMO1 were identified if the distance between a pair of atoms at the interaction interface was smaller than or equal to the sum of their van der Waals radii plus 0.4 Å.

For the comparison of yeast and human Tim17 conformations, distances between all atoms of the aligned residues were calculated, and the protein structure was colored according to root mean square deviation (RMSD) using the colorbyrmsd.py script in the PyMOL Molecular Graphics System, version 3.0 Schrödinger, LLC [32].

## Results

### Existence of two paralogs, TIMM17A and TIMM17B, and their function in health and upon stress

**Fig. 1:**
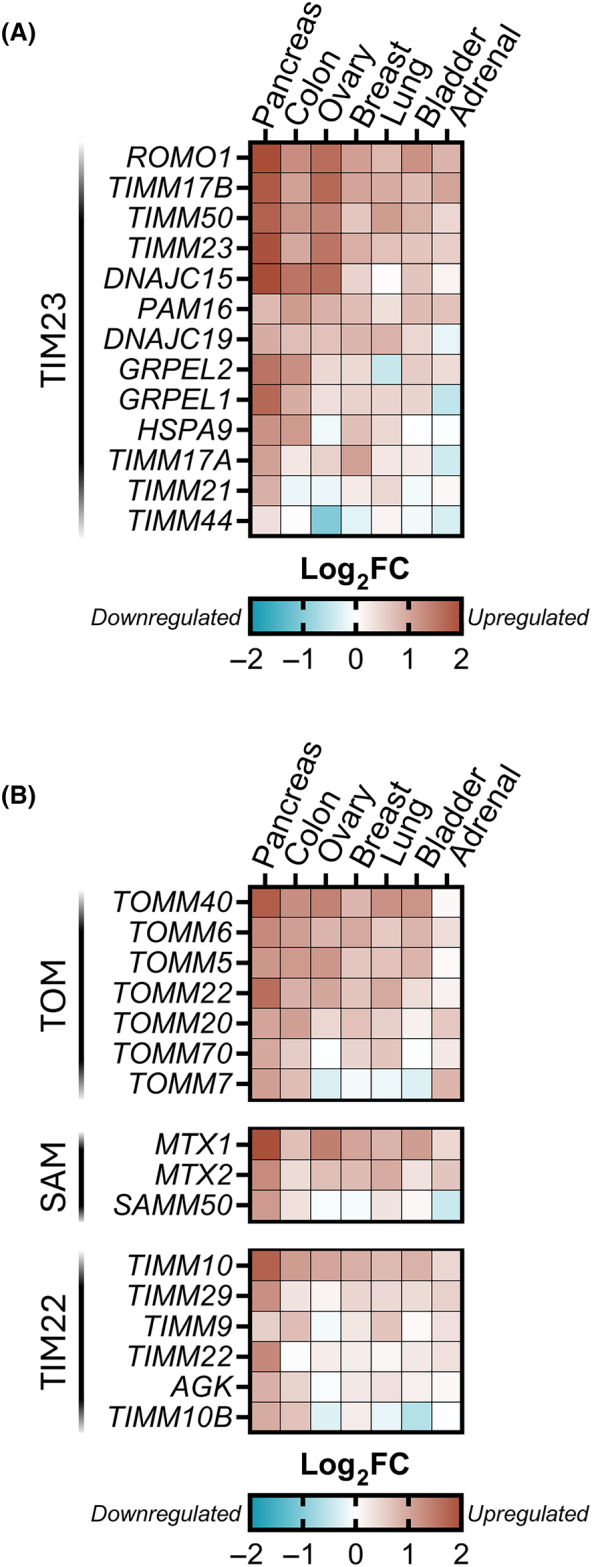
The mRNA levels of different subunits of TIM23, TOM, SAM, and TIM22 complexes in healthy tissue and cancer (A, B). The heatmap depicts log_2_‐fold change (Log_2_FC) in mean gene expression between cancer and normal samples. mRNA‐seq profiles were retrieved from Genevestigator database. Red and blue color intensity indicates gene upregulation and downregulation, respectively.

TIMM17A and TIMM17B paralogs are abundantly expressed, display low tissue specificity, and exhibit 76% sequence identity. The co‐evolution of both proteins shows that although they have very similar primary features, they may differ in their specific functions. Previous research showed that TIMM17B‐containing TIM23 translocase plays a housekeeping role and imports protein clients more readily, whereas TIMM17A‐containing TIM23 plays a secondary role in translocation with a lower import rate [23]. Several studies showed that TIMM17A is degraded in response to various stressors to reduce mitochondrial import and rewire mitochondrial metabolism to adjust to changing conditions [33, 34]. Moreover, the transcriptional upregulation of TIMM17A was implicated in breast cancer [35, 36, 37]. Such a specific behavior of TIMM17A in breast cancer made us question whether the transcription of other components of the TIM23 complex, especially TIMM17B, might be dysregulated in various cancers. Such analyses could pinpoint a specific cancer‐related function of one of the TIMM17 variants. To address this, we queried publicly available gene expression datasets to study cancer‐related changes in mRNA levels of TIM23 complex components. Interestingly, we found that the majority of them were markedly upregulated in tumor tissues compared with control samples from unaffected patients and normal tissues adjacent to the tumor, with TIMM23 and TIMM17B exhibiting the most pronounced shift in expression (Fig. 1A). To check whether the transcriptional upregulation of the subunits of the TIM23 complex is specific in cancer, we extended our study by analyzing the transcript levels of the proteins that build other mitochondrial translocases or insertases, namely translocase of the outer membrane (TOM), mitochondrial sorting and assembly machinery (SAM, also known as TOB) and translocase of the inner membrane 22 (TIM22). Interestingly, most of the analyzed subunits belonging to different translocating systems were transcriptionally upregulated in different cancers (Fig. 1B). We then checked other proteins that belong to various organellar systems such as proteases and peptidases (preprotein cleavage), mitochondrial transcription, complex V of respiratory chain and Fe‐S cluster biosynthesis. The majority of checked proteins were transcriptionally upregulated, which is most likely an outcome of enhanced translocating systems (Fig. S1). Indeed, previous studies reported that mitochondrial biogenesis drives the proliferation of tumor cells and promotes the growth of cancer by providing critical metabolites [38, 39]. Our analysis showed that an increase in transcriptional level in cancer is not a distinctive feature of the TIM23 complex or TIMM17A alone, but there is a general increase in transcript numbers of mitochondrial proteins.

### Structural conservation of the core TIM23 between human and yeast

**Fig. 2:**
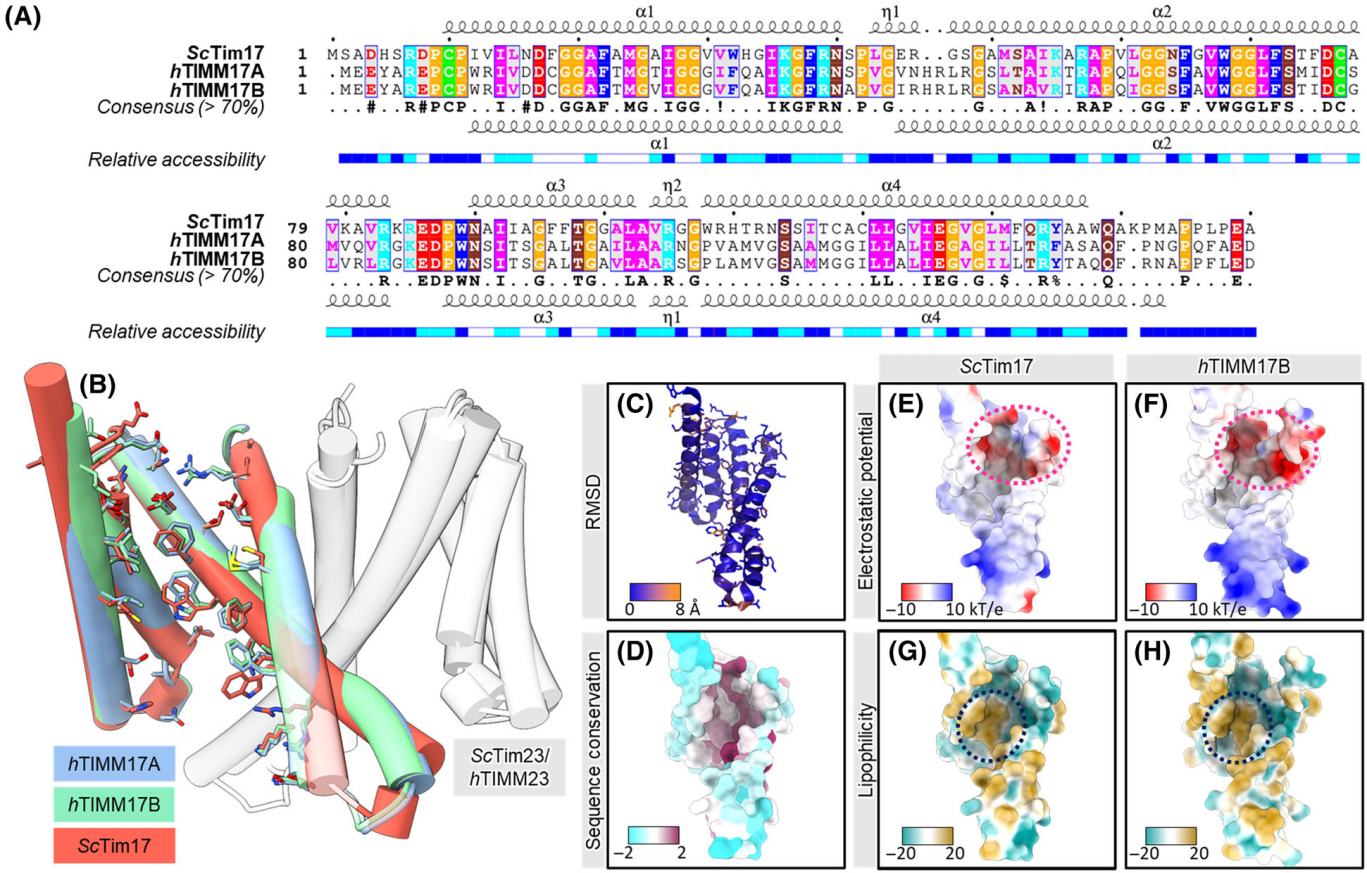
Comparison between yeast Tim17 and its human orthologs TIMM17A and TIMM17B. (A) Alignment of the yeast (ScTim17) and human (hTIMM17) orthologs of Tim17 with amino acid residues colored according to their chemical properties. (B) Superimposition of cryo‐EM structures of yeast Tim23‐Tim17 heterodimer (PDB ID 8SCX) and the AlphaFold predicted structures of human heterodimer built of TIMM23 and TIMM17A or TIMM17B. Only the regions predicted with high confidence (AlphaFold pLDDT score of 70 and above) are shown and were used for the comparisons. Side chains of the residues forming the lateral cavity of Tim17 are shown in the stick representation. (C) Yeast Tim17 aligned and colored by RMSD. Dark blue indicates a good alignment, whereas higher deviations are in orange/yellow/red. (D) Yeast Tim17 colored by its protein sequence conservation. The colors correspond to AL2CO (ALignment 2 COnservation) entropy measure with dark purple indicating highly conserved regions, whereas variable regions are in white/cyan [46]. (E, F) Comparison of electrostatic surface potential and (G, H) lipophilicity of ScTim17 (E, G) and hTIMM17B (F, H) which revealed the conserved negatively charged patch (indicated by pink dashed line) at the IMS side of the protein, and hydrophobic patch inside the channel‐forming cavity (indicated by dark blue dashed line). The colors in panels G and H represent molecular lipophilicity potential, where positive potentials (in goldenrod) correspond to more lipophilic (more hydrophobic) areas, negative (in cyan) to less lipophilic (more hydrophilic) areas [47].

Novel cryo‐EM structures of the yeast TIM23 complex and recent advances in computational structural biology prompted us to compare the core TIM23 complex between yeast and humans and human TIMM17A‐ or TIMM17B‐containing TIM23 complexes. TIMM17A and TIMM17B share 43.6% and 43.9% sequence identities, respectively, with yeast Tim17 (Fig. 2A). Comparisons of Tim17 amino acid sequences from a broader spectrum of 16 species, showed that the most diverse is the C‐terminal unstructured region of the protein (starting at residue 139 in yeast Tim17), which was exposed to the IMS (Fig. S2). In agreement with the low confidence of AlphaFold in predicting the C‐terminal part of Tim17 (expressed as low pLDDT scores, Fig. S3), this region remained too flexible to be resolved in the reported cryo‐EM structures of the TIM23 complex [17, 18]. The predicted structure of the human TIM23 core complex showed a nearly identical arrangement of transmembrane regions of TIMM17A‐TIMM23 and TIMM17B‐TIMM23 heterodimers as shown in the cryo‐EM structure of the yeast Tim17‐Tim23 heterodimer (Fig. 2B). We subsequently compared biochemical properties of yeast Tim17 and human TIMM17B, known to build a housekeeping variant of TIM23. RMSDs of distances between Cα atom pairs of human TIMM17B and yeast Tim17 were 0.81 Å on average, and 1.1 Å for the RMSD between all atom pairs, highlighting similarity of the conformation of both proteins (Fig. 2C). The differences were mostly limited to the loop that connects transmembrane helix 1 and 2. Mapping conservation of the amino acid sequence in the Tim17 structure revealed that the most conserved residues were those that form the cavity that is responsible for translocating the substrate polypeptide into the mitochondrial matrix (Fig. 2D). Similar to yeast Tim17, TIMM17B contains an acidic patch that is located at the IMS side of the cavity (Fig. 2E,F). This acidic patch is built of the conserved Asp17, Asp76, and Glu126 (Asp16, Asp77, and Glu127 in humans). Furthermore, we observed a conserved hydrophobic patch that was located deeper inside the cavity in yeast Tim17 and human TIMM17B (Fig. 2G,H). The hydrophobic nature of the channel facilitates the translocation of presequence‐containing proteins. Altogether, we show that yeast Tim17 and human TIMM17A and TIMM17B showed similar protein folds. Moreover, yeast Tim17 and human TIMM17B displayed consistent electrostatic and hydrophobic properties of the channel‐forming cavity.

### Biochemical properties of the channel‐forming cavity of TIMM17A and TIMM17B are similar

**Fig. 3:**
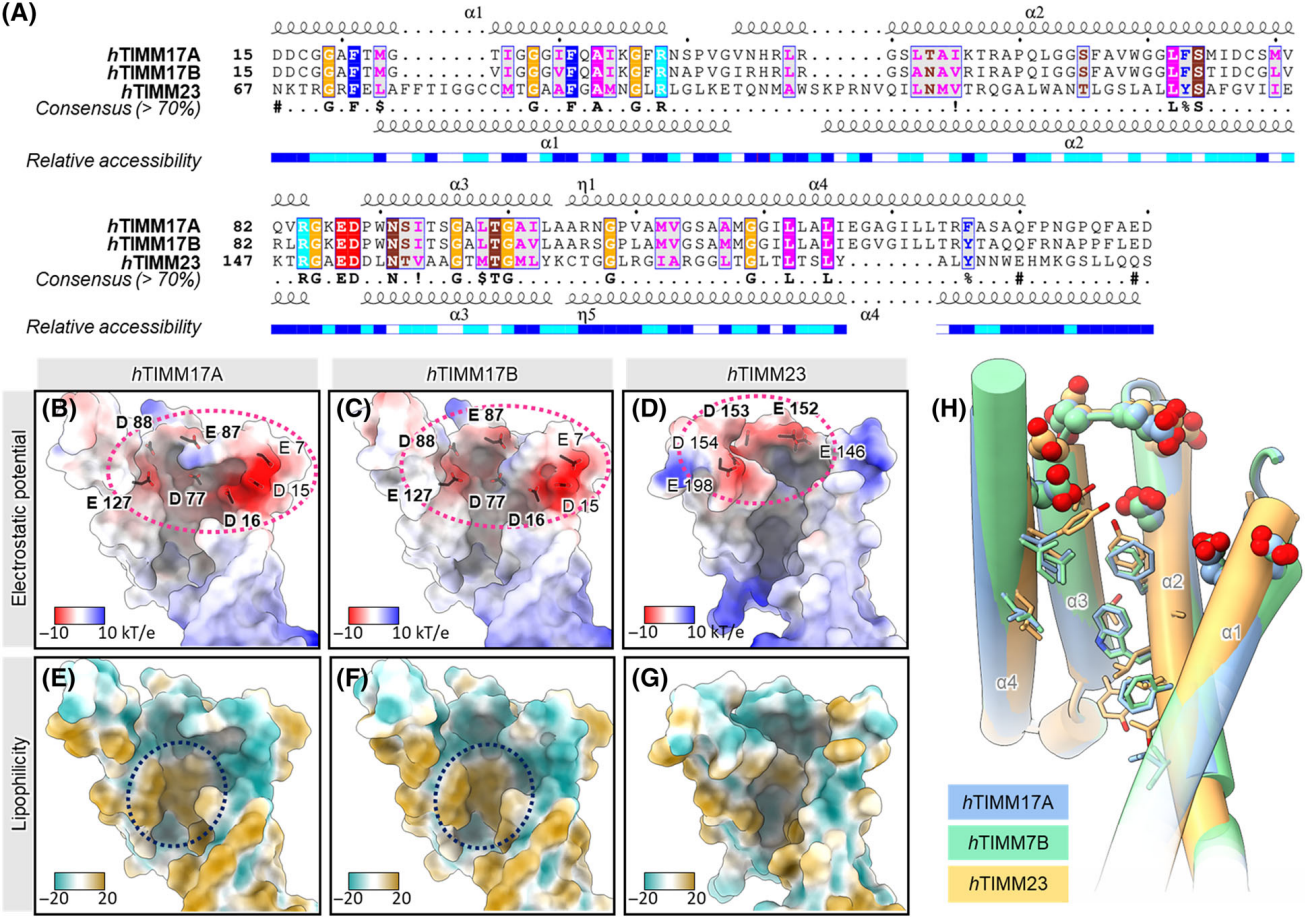
Comparison of human Tim17 family members. (A) Alignment of the human TIMM17A, TIMM17B, and TIMM23 with residues colored according to their chemical properties. (B–D) Comparison of electrostatic surface potential and (E–G) lipophilicity of the transmembrane regions of TIMM17A (B, E), TIMM17B (C, F), and TIMM23 (D, G). The conserved negatively charged patch, and the hydrophobic patch inside the channel‐forming cavity are indicated by pink and blue dashed lines, respectively. The acidic residues at the IMS side of the proteins are shown in stick representation with the name of the conserved residues in bold. Coloring of lipophilicity potential in panels E–G is the same as in Fig. 2G,H. (H) Superimposed transmembrane regions of human TIMM17A, TIMM17B, and TIMM23 with the hydrophobic residues lining the lateral cavity of the proteins shown in stick representation and the acidic residues at the entrance to the cavity in red ball representation.

We subsequently utilized the predicted structures of TIMM17A and TIMM17B to further study their biochemical features in the context of the ability to attract the polypeptide that translocates from the TOM complex. Fielden *et al*. [19] showed that highly conserved negatively charged amino acids of Tim17 (Asp17, Asp76, and Glu126) that are exposed to the IMS are crucial in this process. We found that these negatively charged amino acids are also present in both TIMM17A and TIMM17B and correspond to Asp16, Asp77, and Glu127 (Fig. 3A). We further showed that TIMM17A and TIMM17B contained an IMS‐exposed acidic patch, analogous to the yeast Tim17 (Fig. 3B,C). This indicates that the mechanism of import through the cavity that is formed by Tim17 is conserved between yeast and humans. Additionally, higher eukaryotes possess another negatively charged amino acid residue, Asp15 or Glu15, that precedes highly conserved Asp16 (corresponds to Asp17 in yeast). Asp15/Glu15 also localize to the acidic patch that is exposed to the IMS and hence increase the negative surface charge at the entry to the TIM23 complex. This in turn may modulate the affinity toward presequences of the translocating proteins. We also identified the presence of negative charges at the human TIMM23 IMS‐exposed part (Fig. 3D). However, recent studies in yeast negated their significance for protein import because their mutations did not affect yeast viability [19]. We further analyzed cavities that were formed by TIMM17A and TIMM17B for their potential to perform the translocation of polypeptides. Sim *et al*. [17] found that hydrophobic and aromatic amino acids that line the cavity of Tim17 are highly conserved, supporting its function in protein translocation, whereas the cavity of Tim23 is more variable. We found that the inner surface of the cavity in TIMM17A and TIMM17B was also lined with hydrophobic residues (Fig. 3E,F). This indicates that passage of the amphipathic presequence in both yeast and humans is driven by hydrophobic interactions. We also found that the cavity that is formed by TIMM23 was still lined mostly with hydrophobic and aromatic residues, but it appeared to have an overall less hydrophobic nature than cavities of TIMM17A and TIMM17B (Fig. 3G). Thus, although, TIMM17A and TIMM17B share similar fold and biochemical features with TIMM23 (Fig. 3H), both TIMM17 variants, similar to yeast Tim17, would likely display higher affinity toward the presequence than TIMM23.

### Conservation of cysteine residues in the core of the TIM23 complex

**Fig. 4:**
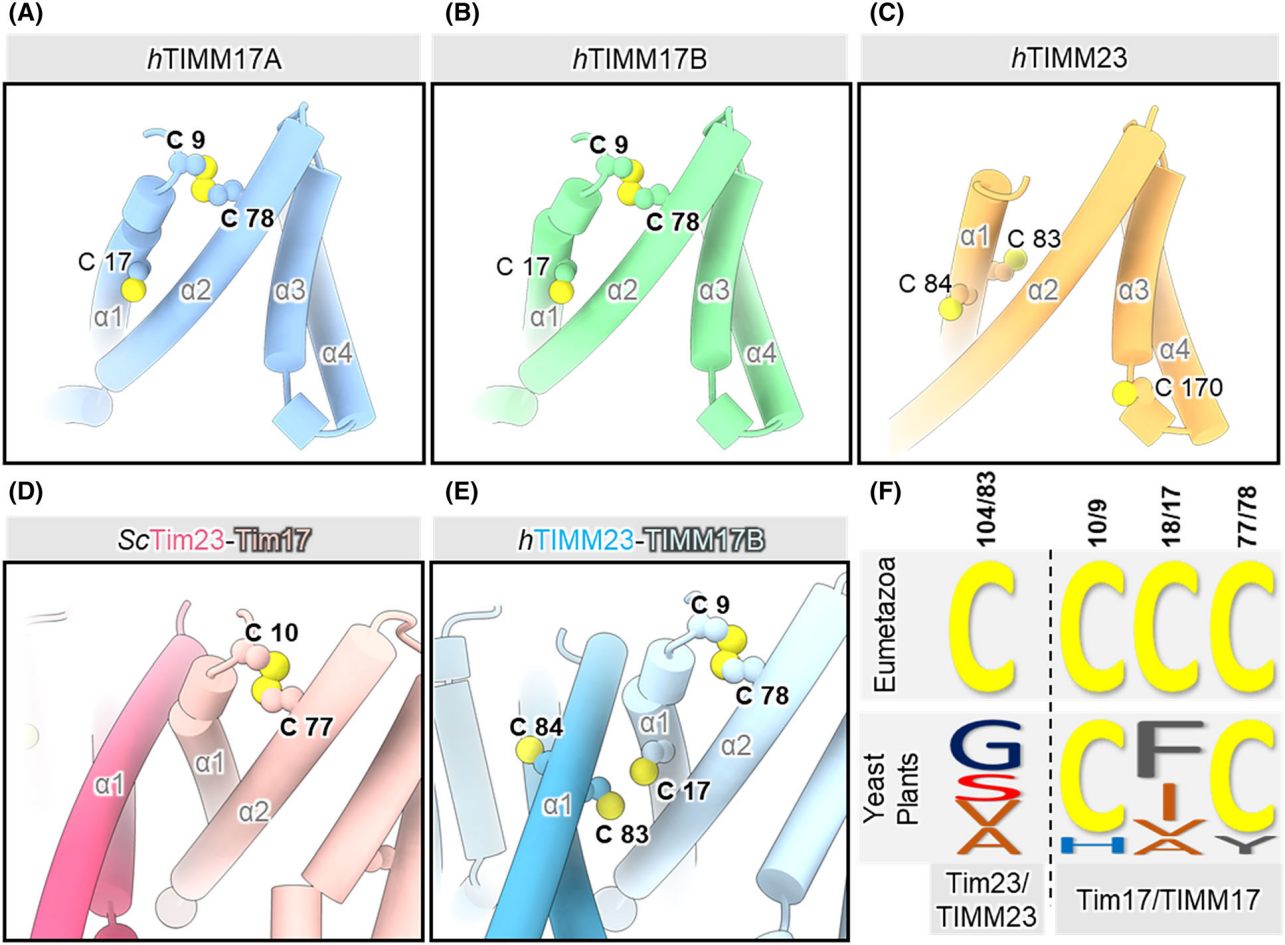
Conservation of cysteine residues and disulfide bonds at the core of the TIM23 complex. (A, B) Conserved pair of Cys9 and Cys78 (corresponding to Cys10 and Cys77 in yeast) forms functionally important intramolecular disulfide bond in both human TIMM17A and TIMM17B that links the transmembrane helix 1 (α1) and 2 (α2). (C) The intramolecular disulfide bonds are not present in the structurally similar human TIMM23. (D) Apart from the disulfide bond formed by the conserved Cys10 and Cys77 residues in Tim17, no other disulfide bridges were observed in the yeast Tim23‐Tim17 heterodimer. (E) In contrast, the Cys17 in the human TIMM17A/B and the Cys83 in the human TIMM23 are located in close vicinity, which could result in an intermolecular disulfide bond formation in certain conformations of the TIMM23 complex. (F) Conservation of the TIM23 key cysteine residues in different branches of the Eukaryota domain. The size of the single letter amino acid code reflects the prevalence of the particular amino acid residue at the particular position in the sequence denoted above on the top of the panel (according to yeast/human sequence of the protein). Both, Cys17 in TIMM17A and TIMM17B and Cys83 in TIMM23 are conserved in Eumetazoa and appeared simultaneously in the course of evolution.

Disulfide bridges are covalent bonds that form between thiol groups of two cysteine residues in the oxidative folding process. Previous studies identified the presence of a single disulfide bond in Tim17 in yeast and TIMM17B in human cells [22, 40]. Moreover, the exact cysteine residues that are involved in the formation of disulfide bonds were pinpointed to positions Cys10 and Cys77 in yeast and Cys9 and Cys78 in humans [22, 40]. The functional and structural importance of this single disulfide bond in Tim17 is also exemplified by the high conservation of cysteine residues that are involved in its formation across eukaryotes (Fig. S2). Because the cysteine residues in both TIMM17A and TIMM17B are conserved, we checked whether the same cysteine residues can form a disulfide bond in TIMM17A. Our computational analysis showed that a disulfide bond highly likely forms within TIMM17A between Cys9 and Cys78 as in TIMM17B (Fig. 4A,B). Next, we focused on cysteine distribution in TIMM23. In agreement with previous studies, we did not predict any intramolecular disulfide bonds (Fig. 4C) [40]. We further showed a lack of an intermolecular disulfide bond between Tim17 and Tim23 in yeast (Fig. 4D). Interestingly, we observed the possible formation of an intermolecular disulfide bond between TIMM17A and TIMM23 and between TIMM17B and TIMM23 (Cys17 in TIMM17A and TIMM17B; Cys83 in TIMM23) (Fig. 4E). The distance between Cα atoms of TIMM17A/B Cys17 and TIMM23 Cys83 was 7.6 Å, which is in the upper range for disulfide‐bonded cysteine residues in experimentally determined structures [41]. However, assuming that the complex remains dynamic to perform its translocase function, the two cysteine residues in certain conformations would possibly be located even closer. Although Cys10 and Cys77 in yeast (and Cys9 and Cys78 in humans) are conserved, the cysteine residue at position 17 only appears in the Eumetazoa, sub‐kingdom of Animalia. Interestingly, the Cys83 of TIMM23 appeared at the same time as Cys17 in TIMM17A/B in the course of evolution (Fig. 4F). This tempts us to speculate that there is structural and functional importance of this cysteine pair between TIMM17A/B and TIMM23 across the Eumetazoa sub‐kingdom. Altogether, using computational tools, we show that the presence of an intramolecular disulfide bridge in Tim17/TIMM17A/TIMM17B is conserved between yeast and humans.

### Lateral cavity of human TIMM17A/TIMM17B is sealed by ROMO1 to form a translocating channel across the inner mitochondrial membrane

**Fig. 5:**
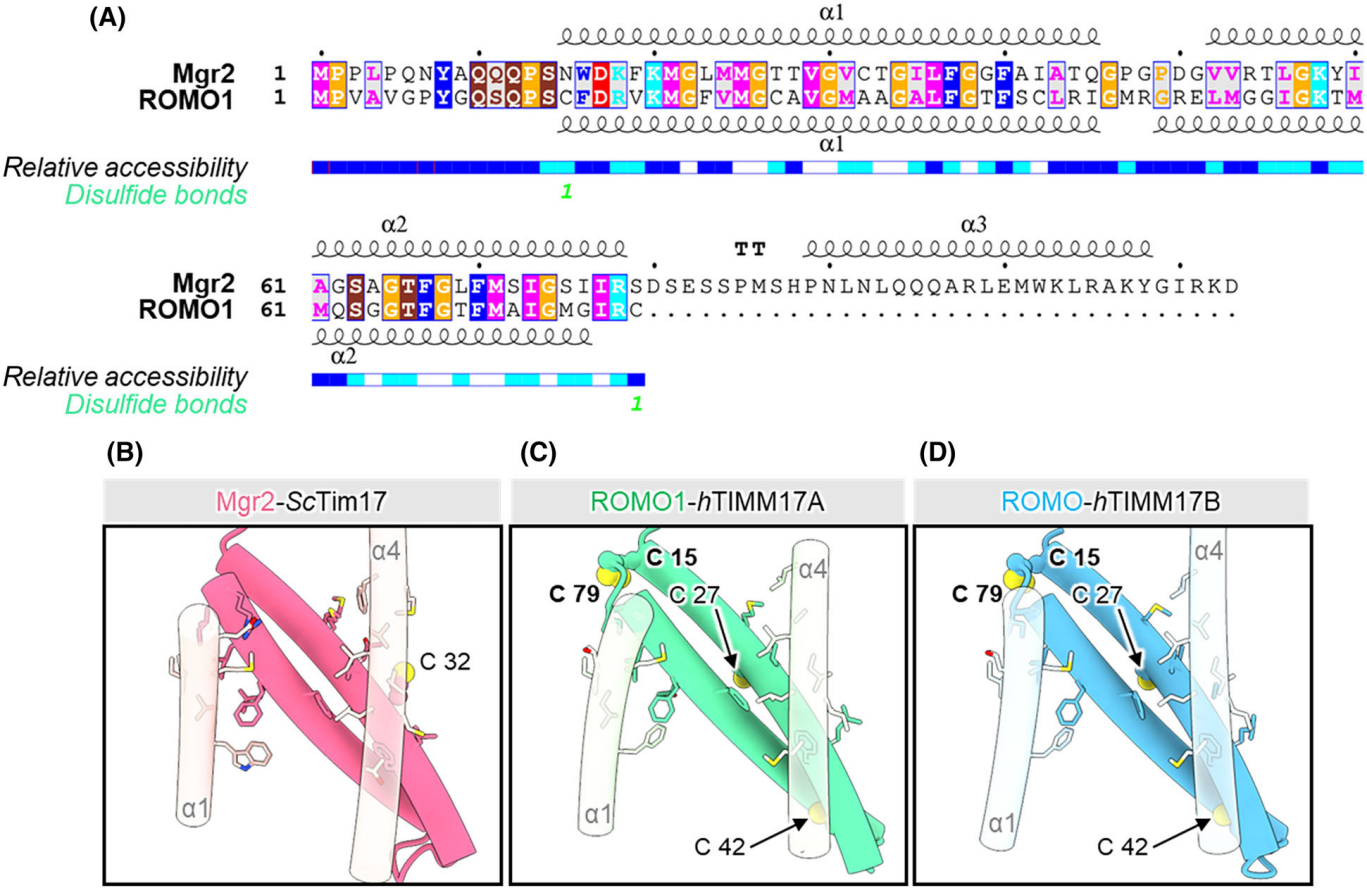
Human ROMO1 (yeast Mgr2) closes the lateral cavity formed by human TIMM17A/TIMM17B. (A) Sequence alignment of Mgr2 and ROMO1 showing conservation of the chemical properties of the transmembrane helices in yeast and human homologs. (B–D) Molecular details of the interface between yeast Tim17 and Mgr2 (B) and human ROMO1 and TIMM17A/B (C, D). The side chains at the interface are shown in stick representation. The cysteine residues that form the disulfide bond linking both of the transmembrane helices of ROMO1 are shown in ball representation.

Mgr2 protein is the latest subunit of the TIM23 complex that was identified in yeast. It is responsible for TOM‐TIM23 coupling, the recruitment of Tim21 to the TIM23 complex, and association of the core TIM23 with respiratory complexes [42]. Subsequent studies demonstrated the importance of Mgr2 in the quality control of mitochondrial precursor proteins to be targeted to the inner membrane [20]. Interestingly, recent structural studies in yeast show that Mgr2 closes the cavity that is formed by Tim17 to shield the translocating precursor from the environment of the lipid bilayer [17, 18]. Such a strategic position in the TIM23 complex and the close proximity to a translocating protein facilitate all of the aforementioned functions of Mgr2. ROMO1 is a human homolog of yeast Mgr2. It was initially found to be a redox‐regulated protein with an important role in mitochondrial fusion and cristae morphology [43]. Later, ROMO1 was reported to be a component of human TIM23, especially critical for the biogenesis of YME1L, an inner membrane protease [44]. Both, Mgr2 and ROMO1 display high amino acid sequence similarity and are built of two transmembrane domains (Fig. 5A). Sim *et al*. [17], using AlphaFold, showed that ROMO1 closes a cavity that is formed by TIMM17B. To further decipher possible differences or similarities between TIMM17A and TIMM17B, we modeled the TIMM17A‐containing TIM23 complex and found that interactions between Mgr2 and Tim17 in yeast and TIMM17A and TIMM17B with ROMO1 in humans are highly similar (Fig. 5B–D). The formed interactions between the proteins rely on hydrophobic interactions between membrane‐exposed residues on transmembrane helix 1 and 2 of Tim17/TIMM17A/TIMM17B and those on outside edges of the harpin formed by the two Mgr2/ROMO1 transmembrane helices. Additionally, we found that a structural conformation of ROMO1, when incorporated into the TIM23 complex, facilitates the formation of an intramolecular disulfide bond between its Cys15 and Cys79 residues, a feature that is absent in yeast Mgr2. The presence of these highly reactive cysteine residues is critical for ROMO1’ function as a redox‐switch protein [43]. However, its functional implications with regard to its role in protein translocation remain to be elucidated. In summary, using structure prediction tools, we show that ROMO1 can close the lateral cavity formed by either TIMM17A or TIMM17B, indicating that the interaction between these proteins is crucial for protein translocation in both yeast and humans.

## Discussion

The first cryo‐EM structures of the core TIM23 complex of yeast were very recently published, which revolutionized our view of mechanisms of protein translocation via this translocase [17, 18, 45]. Previous findings indicate that Tim23 participates in the formation of an aqueous channel [7, 10, 11, 12, 13, 14, 15, 16]. However, recent structural data suggest that Tim23 plays a rather architectural role. Seemingly conflicting data may actually be an indication for dynamics and even the existence of twin conducting channels formed by Tim17 and Tim23. Interestingly, such a suggestion was made before [13]. Future studies could reveal whether at any circumstances Tim23 functions as a protein‐conducting channel.

There are two orthologs of yeast Tim17 in human cells, TIMM17A and TIMM17B. Both were shown to build functional complexes with TIMM23 [23]. However, neither translocase variant has been characterized structurally. Here, using computational tools, we demonstrated that the TIM23 core complexes either built of TIMM17A or TIMM17B are nearly identical. Moreover, comparisons with structures that were recently obtained from yeast, revealed high structural conservation of the core TIM23 complex between yeast and humans. We subsequently showed that biochemical features of TIMM17A and TIMM17B (the presence of the conserved negatively charged amino acid, hydrophobic residues lining the lateral cavity, and the disulfide bridge) are shared between both paralogs and yeast Tim17. We further showed that ROMO1, a homolog of yeast Mgr2, can close the lateral cavity formed by both TIMM17A and TIMM17B.

In summary, two conclusions can be drawn from our study: (a) the core TIM23 complex is structurally conserved between humans and yeast and (b) the TIMM17A‐ and TIMM17B‐containing complexes are nearly identical. The second conclusion poses yet another question. What is the basis of functional differences between both translocase variants in humans, because they are structurally alike? A possible answer is the presence of specific regulatory factors that differentially impact both versions of the translocase. Intriguingly, recent research identified a protein (ovarian cancer immuno‐reactive antigen domain containing 1 [OCIAD1]), that can specifically control levels of TIMM17A‐containing TIM23 (unpublished data). OCIAD1 depletion led to a reduction of the TIMM17A version of the translocase, whereas the TIMM17B version was unchanged. Both translocases can be differentially regulated, raising questions of how exactly such regulation is exercised and what other proteins play a role in this mechanism.

## Conflict of interest

The authors declare no conflict of interest.

### Peer review

The peer review history for this article is available at https://www.webofscience.com/api/gateway/wos/peer‐review/10.1002/2211‐5463.13840.

## Author contributions

Data generation and analysis was performed by KKM, PD and AW. Data interpretation was done by all the authors. All the authors contributed to writing of the manuscript. The study was designed by AC.

## Supporting information


**Fig. S1.** The mRNA levels of different proteins that belong to various mitochondrial systems, preprotein cleavage, mitochondrial transcription, complex V (OXPHOS) and Fe‐S cluster biosynthesis in healthy tissue and cancer.

**Fig. S2.** Multiple sequence alignment of Tim17/TIMM17 homologs.

**Fig. S3.** Quality of the AlphaFold predicted core of the human TIM23 complex.

**Table S1.** Data sources used in gene expression analysis.

**Table S2.** UniProt IDs of the sequences of Tim17 from various species used in multiple sequence alignment.

## Data Availability

Gene expression datasets explored in this study are listed in the Table S1. UniProt accession codes for Multiple Sequence Alignment of Tim17/TIMM17 homologs are listed in the Table S2. PDB accession codes of the yeast TIM23 complex structure: 8SCX and 8E1M.
