# Supplementary figures and images for: Structure prediction analysis of human core TIM23 complex reveals conservation of the protein translocation mechanism
Source: FEBS Open Bio. 2024 Jun 4;14(10):1656–67. doi: 10.1002/2211-5463.13840 (PMC11452300; doi:10.1002/2211-5463.13840)

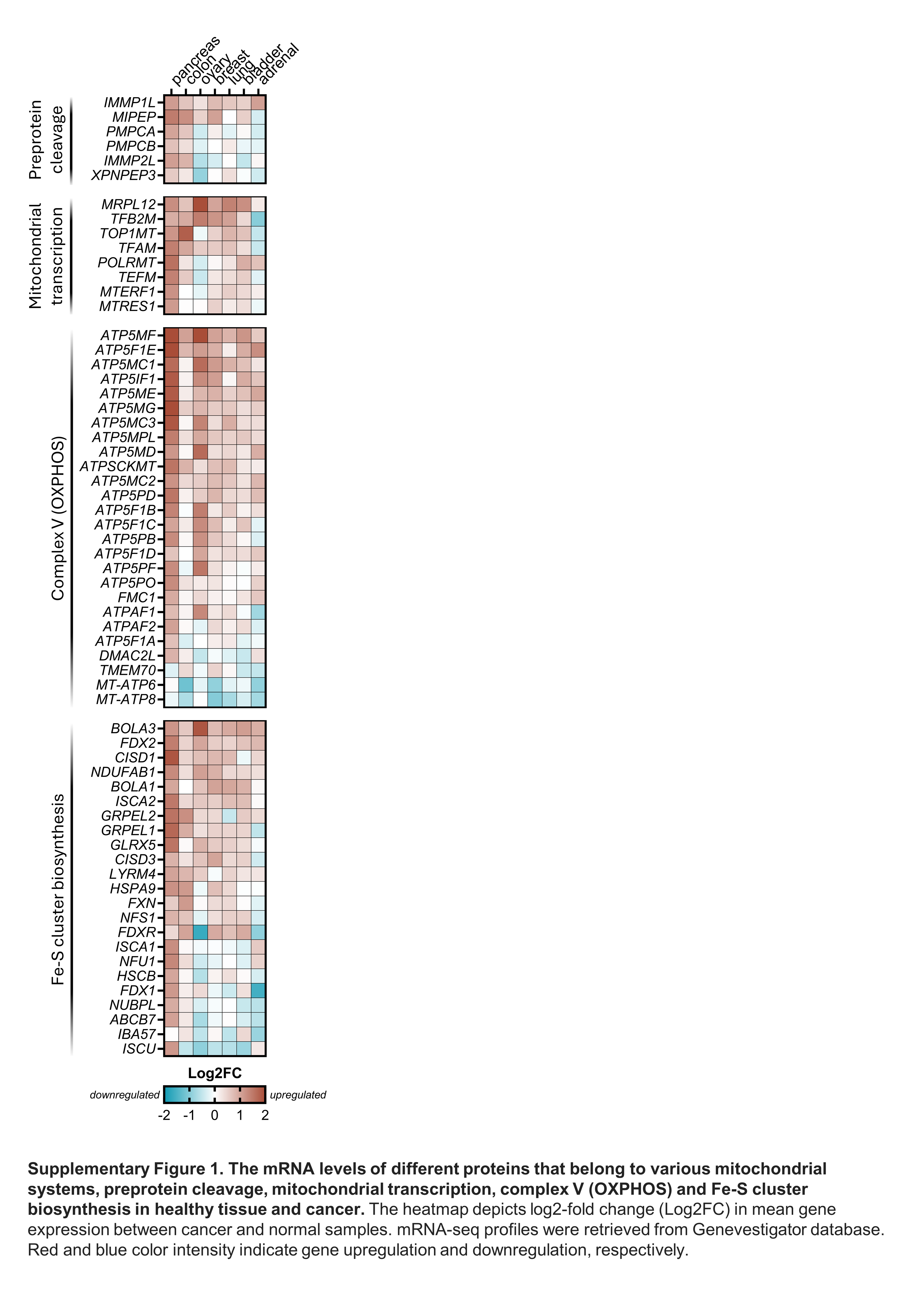

Supplement: Supplementary file 1 — Fig. S1. The mRNA levels of different proteins that belong to various mitochondrial systems, preprotein cleavage, mitochondrial transcription, complex V (OXPHOS) and Fe‐S cluster biosynthesis in healthy tissue and cancer. [file FEB4-14-1656-s003.tif]

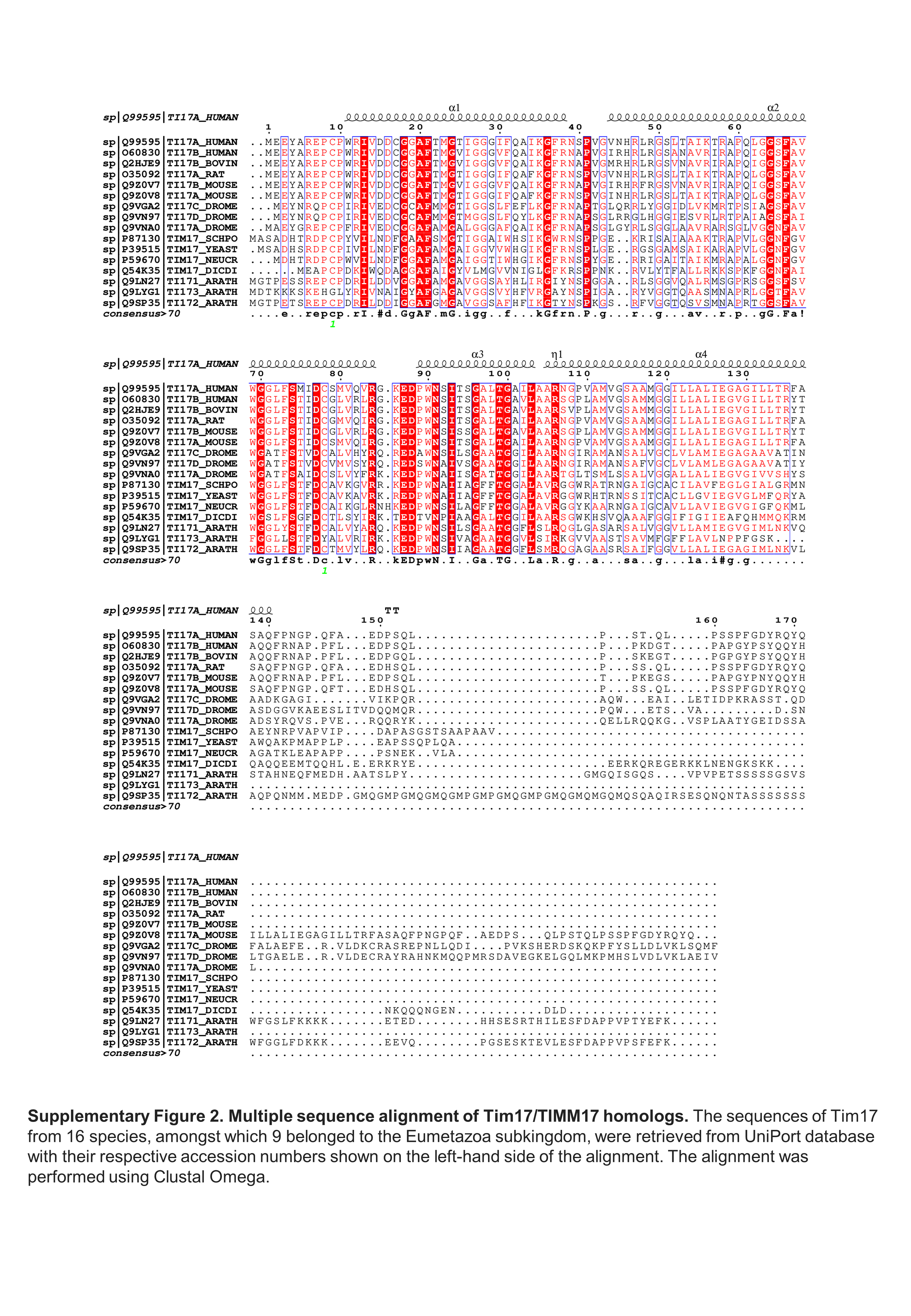

Supplement: Supplementary file 2 — Fig. S2. Multiple sequence alignment of Tim17/TIMM17 homologs. [file FEB4-14-1656-s001.tif]

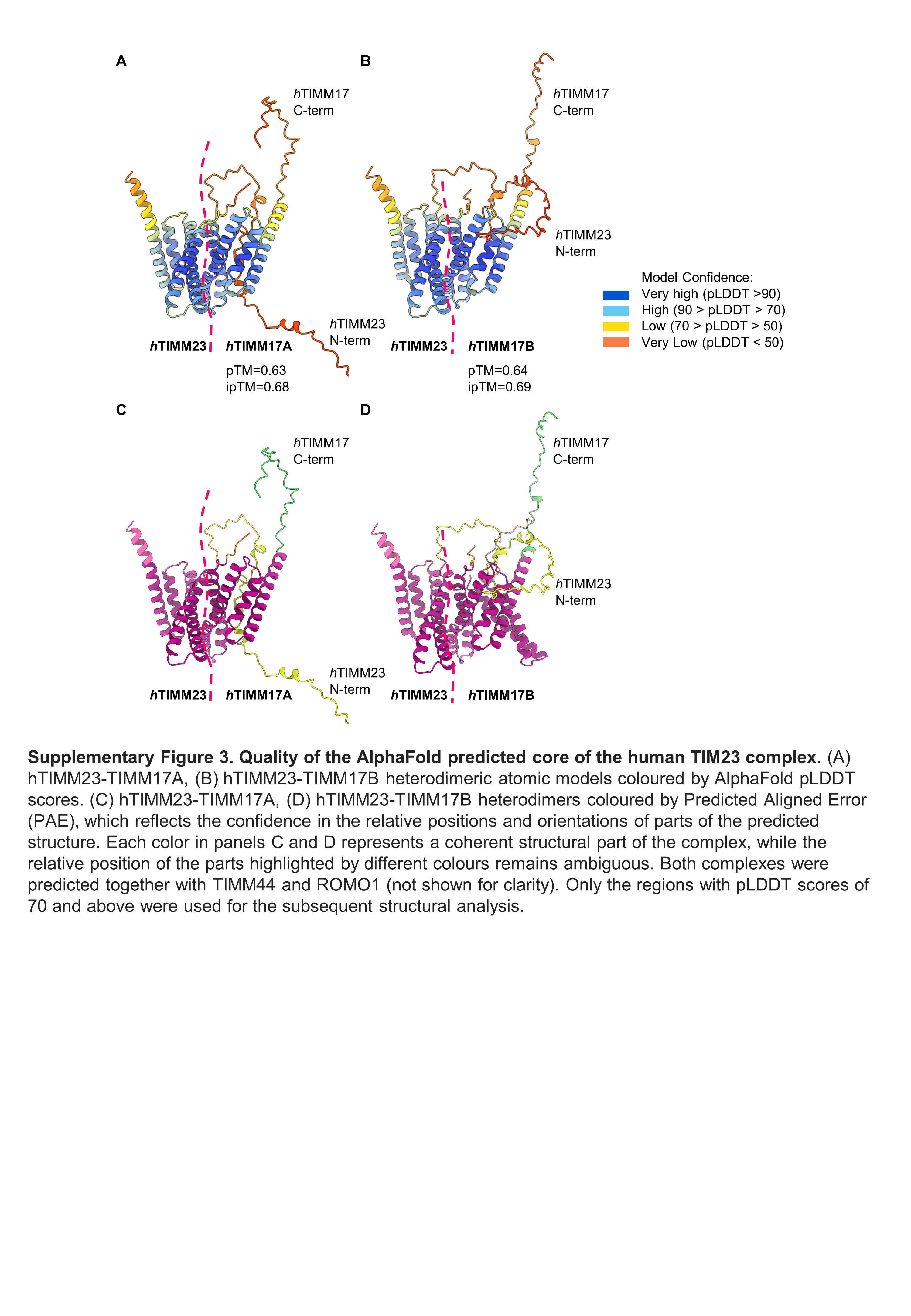

Supplement: Supplementary file 3 — Fig. S3. Quality of the AlphaFold predicted core of the human TIM23 complex. [file FEB4-14-1656-s002.tif]
