# Supplementary material for: Structure prediction analysis of human core TIM23 complex reveals conservation of the protein translocation mechanism
Source: FEBS Open Bio. 2024 Jun 4;14(10):1656–67. doi: 10.1002/2211-5463.13840 (PMC11452300; doi:10.1002/2211-5463.13840)
Supplement: Supplementary file 4 — Table S1. Data sources used in gene expression analysis. [file FEB4-14-1656-s004.docx]

**Supplementary Table 1. Data sources used in gene expression analysis.**

| **Tissue** | | **Experiment ID** |
| --- | --- | --- |
| colon | normal | GSE30611, TCGA-COAD, E-MTAB-1733, SRP127360, GSE83687, GSE165512, SRP012682, GSE65107, GSE235236 |
|  | cancer | TCGA-COAD, GSE74369, GSE50760, GSE76987, E-MTAB-8412 |
| lung | normal | GSE30611, GSE40419, GSE57148, TCGA-LUAD, TCGA-LUSC, phs000178, E-MTAB-1733, E-MTAB-2836, ERP003613, ERP006650, GSE81089, SRP012682, EGAS00001000334, EGAD00001000223, GSE147507, GSE155241 |
|  | cancer | EGAS00001000708, EGAD00001003801, EGAS00001000925, EGAD00001001244, EGAS00001000334, EGAD00001000223, GSE162945 (subseries of GSE162946), GSE81089, TCGA-LUAD, TCGA-LUSC, phs000178, GSE40419, GSE158403 |
| pancreas | normal | E-MTAB-1733, E-MTAB-2836, ERP003613, ERP006650, TCGA-PAAD, phs000178, SRP012682 |
|  | cancer | TCGA-PAAD, phs000178 |
| breast | normal | GSE30611, GSE58135, TCGA-BRCA, phs000178, SRP012682 |
|  | cancer | GSE58135, TCGA-BRCA, phs000178, GSE139050, GSE51124 |
| ovary | normal | GSE30611, E-MTAB-1733, E-MTAB-2836, ERP003613, ERP006650, SRP012682 |
|  | cancer | GSE188249 (subseries of GSE188250), TCGA-OV, phs000178 |
| adrenal | normal | GSE30611, TCGA-PCPG, phs000178, E-MTAB-1733, E-MTAB-2836, ERP003613, ERP006650, SRP012682 |
|  | cancer | TCGA-ACC, phs000178, TCGA-PCPG, phs000178 |
| bladder | normal | TCGA-BLCA, phs000178, E-MTAB-1733, E-MTAB-2836, ERP003613, ERP006650, SRP012682 |
|  | cancer | TCGA-BLCA, phs000178 |
