# Supplementary material for: Structure prediction analysis of human core TIM23 complex reveals conservation of the protein translocation mechanism
Source: FEBS Open Bio. 2024 Jun 4;14(10):1656–67. doi: 10.1002/2211-5463.13840 (PMC11452300; doi:10.1002/2211-5463.13840)
Supplement: Supplementary file 5 — Table S2. UniProt IDs of the sequences of Tim17 from various species used in multiple sequence alignment. [file FEB4-14-1656-s005.docx]

| **Gene, organism** | **UniProt ID** |
| --- | --- |
| TIMM17A, Homo Sapiens (Human) | Q99595 |
| TIMM17B, Homo Sapiens (Human) | O60830 |
| TIMM17B, Bos taurus (Bovine) | Q2HJE9 |
| Timm17a, Rattus norvegicus (Rat) | O35092 |
| Timm17b, Mus musculus (Mouse) | Q9Z0V7 |
| Timm17a, Mus musculus (Mouse) | Q9Z0V8 |
| Tim17a1, Drosophila melanogaster (Fruit Fly) | Q9VGA2 |
| Tim17a2, Drosophila melanogaster (Fruit Fly) | Q9VN97 |
| Tim17b1, Drosophila melanogaster (Fruit Fly) | Q9VNA0 |
| tim17, Schizosaccharomyces pombe (Fission yeast) | P87130 |
| TIM17, Saccharomyces cerevisiae (Baker’s yeast) | P39515 |
| tim17, Neurospora crassa | P59670 |
| timm17, Dictyostelium discoideum (Social amoeba) | Q54K35 |
| TIM17-1, Arabidopsis thaliana (Mouse-ear cress) | Q9LN27 |
| TIM17-3, Arabidopsis thaliana (Mouse-ear cress) | Q9LYG1 |
| TIM17-2, Arabidopsis thaliana (Mouse-ear cress) | Q9SP35 |

**Supplementary Table 2. UniProt IDs of the sequences of Tim17 from various species used in multiple sequence alignment.**
